# Rapid accumulation and low degradation: key parameters of *Tomato yellow leaf curl virus* persistence in its insect vector *Bemisia tabaci*

**DOI:** 10.1038/srep17696

**Published:** 2015-12-02

**Authors:** Nathalie Becker, Loup Rimbaud, Frédéric Chiroleu, Bernard Reynaud, Gaël Thébaud, Jean-Michel Lett

**Affiliations:** 1 Institut de Systématique, Évolution, Biodiversité ISYEB - UMR 7205 - CNRS, MNHN, UPMC, EPHE. Muséum National d’Histoire Naturelle, Sorbonne Universités, 57 rue Cuvier, CP 50, F-75005, Paris, France; 2 CIRAD, UMR PVBMT, Pôle de Protection des Plantes, 7 chemin de l’Irat, F-97410 Saint Pierre, Ile de La Réunion, France; 3 Montpellier SupAgro, UMR 385 BGPI, F-34398 Montpellier, France; 4 INRA, UMR 385 BGPI, F-34398 Montpellier, France

## Abstract

Of worldwide economic importance, *Tomato yellow leaf curl virus* (TYLCV, *Begomovirus*) is responsible for one of the most devastating plant diseases in warm and temperate regions. The DNA begomoviruses (*Geminiviridae)* are transmitted by the whitefly species complex *Bemisia tabaci*. Although geminiviruses have long been described as circulative non-propagative viruses, observations such as long persistence of TYLCV in *B. tabaci* raised the question of their possible replication in the vector. We monitored two major TYLCV strains, Mild (Mld) and Israel (IL), in the invasive *B. tabaci* Middle East-Asia Minor 1 cryptic species, during and after the viral acquisition, within two timeframes (0–144 hours or 0–20 days). TYLCV DNA was quantified using real-time PCR, and the complementary DNA strand of TYLCV involved in viral replication was specifically quantified using anchored real-time PCR. The DNA of both TYLCV strains accumulated exponentially during acquisition but remained stable after viral acquisition had stopped. Neither replication nor vertical transmission were observed. In conclusion, our quantification of the viral loads and complementary strands of both Mld and IL strains of TYLCV in *B. tabaci* point to an efficient accumulation and preservation mechanism, rather than to a dynamic equilibrium between replication and degradation.

Host-to-host transmission is a major step in virus life cycles. The transmission of the vast majority of plant viruses relies on additional organisms, known as vectors. These transmission strategies have been classified as either non-circulative (in contrast with animal viruses, many plant viruses remain in the mouthparts or foregut of the vectors), or circulative (the viruses cross the gut epithelium and ultimately colonize the salivary glands)^1^.

Successful circulative transmission implies that, after uptake by a vector from an infected plant, the virus has to cross several physical barriers to reach the plant, which involve specific virus–vector interactions^2^,^3^: the gut epithelium barrier between gut lumen and the hemocoel, the salivary gland cells between the hemocoel and the saliva duct. This process results in a delay (called latency) between the acquisition of the virus and its transmission through the saliva.

The circulative transmission mode is divided into two sub-categories depending on whether the virus actually replicates or not during its course through the body of its vector (propagative or non-propagative transmission, respectively). Whilst circulative propagative transmission is the major mode of transmission of arthropod-borne viruses to vertebrates, it concerns only a minority of plant viruses which consist of RNA viruses^1^.

In contrast, circulative non-propagative transmission appears to be restricted to plant viruses. Among plant viruses known - or assumed - to use this mode of transmission, geminiviruses consist of circular single-stranded DNA (ssDNA) of to 2.6 to 3.0 kb, packed into twin-shaped icosahedral particles^4^. Begomoviruses, one of the seven known genera of the family *Geminiviridae*, are transmitted by the whitefly species complex *Bemisia tabaci*^5^. After begomovirus transmission to the plant, a circular viral ssDNA is released from the capsid, converted through complementary strand replication (CSR) to double-stranded DNA (dsDNA) replicative form with the aid of host enzymes, and further processed into covalently closed circular DNA (cccDNA), ultimately forming minichromosomes. For further replication of viral DNA, rolling circle replication (RCR) is initiated by the viral replication-associated protein (Rep). During each replication cycle, RCR produces a newly synthesized ssDNA circle (viral strand)^4^,^6^. The identification of additional forms of DNA, such as heterologous dsDNA linked to cccDNA, revealed a complementary strategy of recombination-dependent replication (RDR), providing a mechanism generating genetic diversity, as well as a spectrum of candidate DNA forms for cell to cell and long distance movement within the plant^7^.

Among begomoviruses, the *Tomato yellow leaf curl virus* (TYLCV), responsible for one of the most devastating plant diseases in warm and temperate regions^8^, is ranked among the 10 most important plant viruses in the world^9^. It has been shown that, after an acquisition access period (AAP) of 48 hours on an infected plant, the Middle East-Asia Minor 1 (MEAM1) and the Mediterranean (MED) cryptic species of *B. tabaci* (the two major invasive species of this cryptic species complex) were able to transmit TYLCV during their entire adult lifetime (up to 31 days after acquisition)^10^. As lifetime persistence periods of TYLCV in the vector raised the question of replication, this aspect was investigated by several scientific groups.

Using different semi-quantitative methods (such as Southern blotting, radionucleotide incorporation and primer extension) on groups of 10 to 50 viruliferous insects, a transient increase in TYLCV DNA was observed in MEAM1 *B. tabaci* after an AAP on TYLCV-infected plants, followed by transfer onto non-host plants. This increase was interpreted as multiplication of TYLCV in its vector^11^,^12^. Moreover, the identification of viral transcripts from both the viral and complementary strand using RT-PCR^12^, real-time RT-PCR^13^, and RNA probes^14^, suggested the existence of a dsDNA replicative form in the insect. Since transovarial transmission is reported to be a feature of propagative viruses^3^, although extensive studies are lacking, this aspect was also investigated for TYLCV. In specific studies, TYLCV was indeed transmitted to plants by the offspring of viruliferous MEAM1 *B. tabaci* whiteflies^13^,^10^. However, in subsequent studies, the progeny of viruliferous females were not infectious even though TYLCV DNA was detected in these insects^15^,^16^,^17^. Taken together, these observations are consistent with a possible replication of TYLCV in its insect vector *B. tabaci*, without any clear evidence for transovarial transmission.

We therefore considered the controversial question of TYLCV replication in its vector worth reviewing in the light of today’s quantitative methods. In the present study, we compared the kinetics of a viral AAP, followed by a post-AAP (feeding on a virus non-host plant), in the invasive MEAM1 *B. tabaci.* Representatives of the two emerging TYLCV strains were used, the Mild (TYLCV-Mld) and the Israel (TYLCV-IL) strains. During AAP followed by post-AAP, we quantified TYLCV DNA in MEAM1 *B. tabaci* at different timescales (0–144 hours or 0–20 days). We first used real-time PCR to monitor both viral and complementary DNA strands, without distinction. Next, anchored real-time PCR enabled us to quantify specifically the complementary DNA strand (used as template of viral replication). According to the models which fitted best to these quantitative data, our main results indicated that even if DNA of both TYLCV strains accumulated exponentially during the AAP, it remained stable during post-AAP. Neither replication nor vertical transmission were observed. Our quantification of the viral loads and complementary strands of both Mld and IL strains of TYLCV in *B. tabaci* thus point to an efficient accumulation and preservation mechanism, rather than to a dynamic equilibrium between replication and degradation.

## Results and Discussion

### Similar exponential accumulation of TYLCV strains in *B. tabaci* during viral acquisition

Previous data concerning TYLCV-IL acquisition by MEAM1 *B. tabaci* had been obtained by semi-quantitative methods (Southern blotting), and indicated a maximal viral load of 6 × 10^8^ viral genomes per insect, reached between 24 h and 48 h of AAP; similar loads were estimated for the related *Tomato yellow leaf curl Sardinia virus* (TYLCSV)^18^,^12^. Using real-time PCR, Ohnishi *et al.* (2009)^19^ obtained a maximum of 4 × 10^8^ TYLCV-Mld genomes per adult MEAM1 *B. tabaci* after an AAP of nine days.

**Figure 1:**
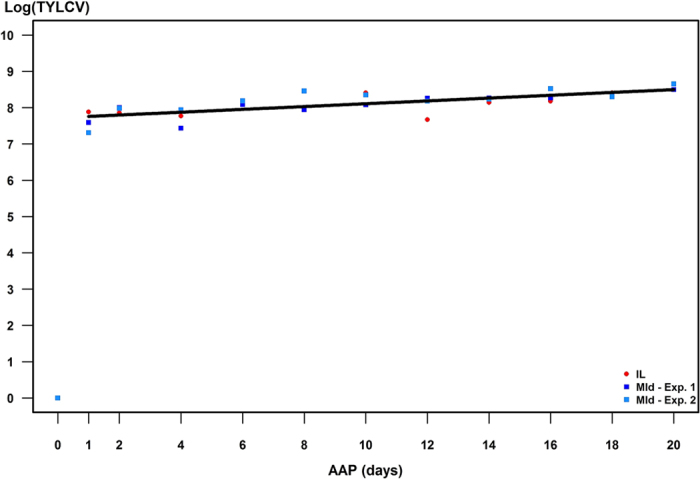
Kinetics of TYLCV-IL and -Mld in *Bemisia tabaci* during a 20-day acquisition access period on agro-infected tomato plants. Each symbol (mean of a duplicate real-time PCR) indicates the mean number of viral genomes per insect, for three independent experiments (one with TYLCV-IL, two with TYLCV-Mld). Intercept of the unique linear regression: 7.72 (Student’s test, p = 1.6×10^−37^). Adjusted R-squared: 0.548.

The average lifespan of MEAM1 *B. tabaci* females on tomato being about 30 days^20^,^21^, we monitored TYLCV acquisition during 20 days of AAP using real-time PCR. Preliminary data from our group indicated no significant difference in the viral load between the two strains (TYLCV-Il and TYLCV-Mld), after an AAP of 72 h in MEAM1 *B. tabaci*^22^. Similarly, no difference was detected between experimental replicates and strains from our present data, (chi2 test, 1 df, p = 0.11 and 0.74, for replicates and strains respectively). We thus used a single linear equation to model how the logarithm of the viral load increased over time. The resulting slope differed significantly from 0 (t-test, p = 1.77 × 10^−6^) and reached more than 10^8^ viral genomes per insect without a detectable plateau (Fig. 1), suggesting interestingly that the carrying capacity of the vector is not reached during this period (representing two thirds of its average lifespan). On an arithmetic scale, this implies an exponential increase in the viral load over the 20 days of AAP.

**Figure 2:**
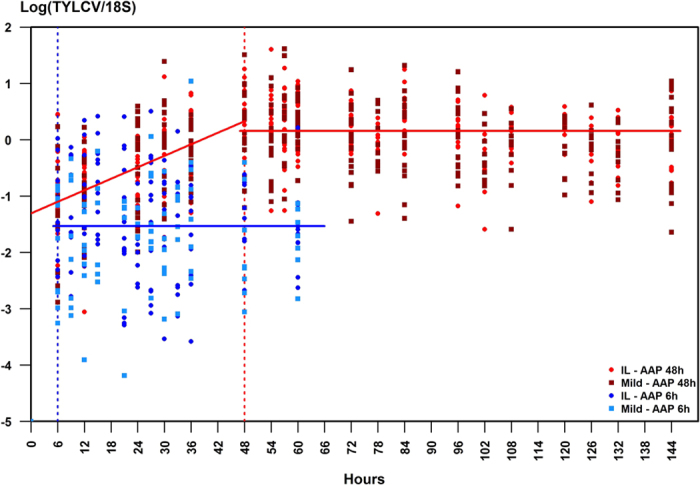
Kinetics of TYLCV-IL and -Mld in *Bemisia tabaci* maintained on virus non-host plants following a viral acquisition access period (AAP) of 6 h and 48 h. Temporal changes in the logarithm of TYLCV real-time PCR values normalized by the *B. tabaci* 18S gene. Each dot represents the mean of a triplicate (circles for TYLCV-IL, squares for TYLCV-Mld, blue for 6 h of AAP, red for 48 h of AAP); the solid line represents the common model: blue for 6 h of AAP, red for 48 h of AAP.

A normalised real-time duplex PCR enabled accurate assessment of the dynamics of viral DNA accumulation during a 48 h AAP in individual insects. Based on the Akaike information criterion (AIC)^23^, the exponential model was used to fit the data (exponential model: AIC = 450.3; power model: AIC = 455.8; linear model: AIC = 481.9). The strain effect was not significant either for the slope (Fisher test with 1 and 1 df, p = 0.91), or for the intercept (Fisher test with 1 and 1 df, p = 0.624). Thus, the common model describing the accumulation dynamics of the two strains is 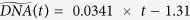, which on an arithmetic scale and expressed in absolute quantities translates into DNA_TYLCV_(*t*) = 1.96×10^5^ × e^0.077×t^ (t being expressed in hours in both cases). As shown in Fig. 2, the viral load increases in the same way for the two strains, reaching 8.45×10^6^ viral genomes per insect after 48 h of AAP (Figure S1 and Table S1). Altogether, reinforcing the results shown in Fig. 1, IL and Mld strains appear to be acquired by *B. tabaci* in a similar way.

In comparison, the viral load of the circulative non-propagative *Maize streak virus* (MSV, *Mastrevirus*, *Geminiviridae*) in the leafhopper vector *Cicadulina mbila*, quantified by real-time PCR at similar time points during the AAP, showed linear accumulation and reached less than 10^6^ copies per insect after 12 days of AAP^24^. The best-fitting exponential model of TYLCV accumulation during the AAP might be consistent with virus replication in its insect vector. Alternatively, the viral load could also increase in the sieve tubes of the host plant during growth and development of newly infected leaves, representing sources of viral particles and nucleoproteins^25^, or during vector feeding, thus accounting for an exponential accumulation during the AAP within the insect without replication in its insect vector. As a first example supporting this hypothesis, co-localized TYLCV coat protein and tomato HSP70 were described in aggregates of increasing size during the course of TYLCV infection in tomato plants, in cells associated with the vascular system^26^. In another example, *Cauliflower mosaic virus* (CaMV, *Caulimoviridae*) was found to be rapidly redistributed after a signal initiated by aphid feeding throughout the plant cell, thus enhancing acquisition^27^. Such responses might contribute to the accumulation of TYLCV viral nucleoproteins in the phloem sap during vector feeding.

### Important stability of the TYLCV load for IL and Mld strains during post-acquisition access periods after 6 to 48 hours of AAP

Among known propagative viruses, 10- to 100-fold increase in viral load has been observed using quantitative PCR during the first day post-AAP with the *Chikungunya virus* (CHIKV) transmitted by the mosquito *Aedes albopictus*^28^, and a 10-fold increase between one and five days post-AAP for the *Raspberry latent virus* (RpLV) transmitted by the aphid *Amphorophora agathonica*^29^. Supporting the hypothesis of TYLCV-IL replication in MEAM1 *B. tabaci*, previous semi-quantitative methods reported transient viral accumulation during the first hours post-AAP^11^,^12^. More precisely, following 12 h of AAP on pools of five insects, a two-fold increase of viral loads was reported in *B. tabaci* during the first 100 h on non-host plants, subsequently remaining stable from 100 to 180 h^11^.

If the exponential increase in viral load we observed during 48 h of AAP (Fig. 2), as well as the monitoring of a continuous increase during 20 days of AAP (Fig. 1), were due to viral replication in the insect vector, we would expect an increase in the viral load post-AAP. Using normalised real-time PCR, we thus studied TYLCV kinetics in individual insects during 54 h and 96 h post-AAP, after AAPs of 6 h and 48 h respectively (Fig. 2, Table S1 and Figure S1).

No significant increase could be detected in our experiments, in which we assessed more than 40 individuals for each time point (10 insects analysed individually per group and per strain) using normalized quantitative PCR. The DNA load of TYLCV-Mld and -IL was best represented by a constant model which depended only on the duration of the AAP (the effects of time post-AAP, strain and experiment were not significant). The common model for the two strains was 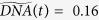 after an AAP of 48 h (Fig. 2, red line) and 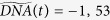 after an AAP of 6 h (Fig. 2, blue line). Figure S1 depicts absolute viral loads per insect on an arithmetic scale: after an initial (although non-significant) drop following the end of AAPs of 6 h and 48 h, a mean value of respectively 56,500 and 5.67×10^6^ viral genomes per insect was maintained until the end of the monitoring period (Table S1).

As a conclusion from the undetectable viral load increase post-AAP, we suggest that TYLCV does not replicate in its insect vector, or at the most that replication is not significant enough to be detected in our experiments.

The remarkable stability of the viral load could be either the result of preservation mechanisms without replication, or of a dynamic equilibrium between virus replication and degradation.

### Low viral degradation throughout the 20 days following the acquisition access period

To gain further insight into the stability of the viral load during a long period on a viral non-host plant, viral DNA was monitored with real-time PCR on MEAM1 *B. tabaci* during 20 days post-AAP, following acquisition on TYLCV-infected plants as described in Materials and Methods. Viral DNA was extracted from groups of whole insects, as well as from dissected insect parts, i.e. abdomen, thorax (including the salivary glands^30^), head (with the salivary canal in the stylet bundle^31^) and haemolymph. All the adjusted R-squares of the corresponding linear regressions were above 0.99. The significance of the parameters of the models (DNA load over time) was assessed with ANOVA.

In whole insects, the viral load decreased slightly during post-AAP in one experiment performed with the Mld strain (Exp. 2, significant negative slope estimated at −0.028), whilst the viral loads in the two other experiments did not decrease significantly (Table 1). In the abdomen, TYLCV-Mld significantly decreased through time (post-AAP, Exp. 1 and 2, Table 1). In the haemolymph, TYLCV-IL increased significantly through time (post-AAP, Table 1, Student’s t test p=0.045); this increase may be linked to continuous viral translocation into the haemolymph. Whatever the strain or the experiment, no other insect part was associated with significant TYLCV increase or decrease post-AAP. In dissected insect parts from 20-day AAP experiments, TYLCV increased significantly (AAP, Table 1), similarly to TYLCV in whole insects (Fig. 1). Our quantitative results point to the persistence, or to a low rate of degradation, of the TYLCV-IL and TYLCV-Mld strains in MEAM1 *B. tabaci* after the AAP. Altogether, the presence of TYLCV-Mld and TYLCV-IL in the thorax (including the salivary glands), abdomen (gut), head (including the salivary canal) and haemolymph during the entire post-AAP period is consistent with their known circulative transmission and the persistence of their transmission capacity.

To assess relative differences between each insect part, the intercepts of the linear regressions of TYLCV loads were compared within each experiment, and ranked according to significance groups (Table 1). The analysis revealed two to four significance groups in each of the experiments, compatible with the relative size of the abdomen, thorax and haemolymph. In particular, in all experiments performed with the Mld strain, the TYLCV loads of the head belonged to the lowest-value group. On the contrary, for all the experiments performed with the IL strain, the TYLCV loads of the head were significantly above the lowest-value group. This higher proportion of viral DNA of TYLCV-IL in the head requires further investigation. TYLCV-interacting proteins (for review Czosnek *et al.*, 2007) might contain Mld- or IL- specific interaction domains, responsible for the differences observed in this study.

Taken together, our long-term persistence study (together with previous semi-quantitative studies which have shown that TYLCV-IL and TYLCSV are retained throughout the life of the whitefly, and remain transmissible for a long period after acquisition^10^,^12^,^19^,^32^,^33^), indicate long-term stability of the viral load in *B. tabaci*. Our quantification of the viral loads and DNA complementary strands of both Mld and IL strains of TYLCV in MEAM1 *B. tabaci* during non-acquisition access periods points to an effective preservation mechanism, rather than to substantial replication to compensate for degradation. Our results reinforce the “transmissible reservoir” hypothesis in the case of the circulative non-propagative transmission mode of viruses by insect vectors^10^,^12^,^18^,^32^,^34^. A repertoire of *B. tabaci* chaperonins^35^ might be involved in this long-term conservation (HSP70^36^,^37^ or BtHSP16^38^) and transmission (GroEL chaperonins encoded by secondary endosymbionts of *B. tabaci*, such as *Hamiltonella sp.*^39^ or *Rickettsia sp.*^34^).

### Detection of the TYLCV complementary DNA strand during and after viral acquisition access periods

According to the RCR mechanism, if TYLCV replicates in *B. tabaci*, neosynthesis of a complementary strand is required. To detect this strand, we designed an anchored real-time PCR to specifically amplify the TYLCV complementary strand in *B. tabaci*. Insects were processed independently during 20 days, either during a constant AAP to monitor the presence of the TYLCV complementary strand, or most importantly after 3 days AAP to detect its potential neosynthesis.

**Figure 3:**
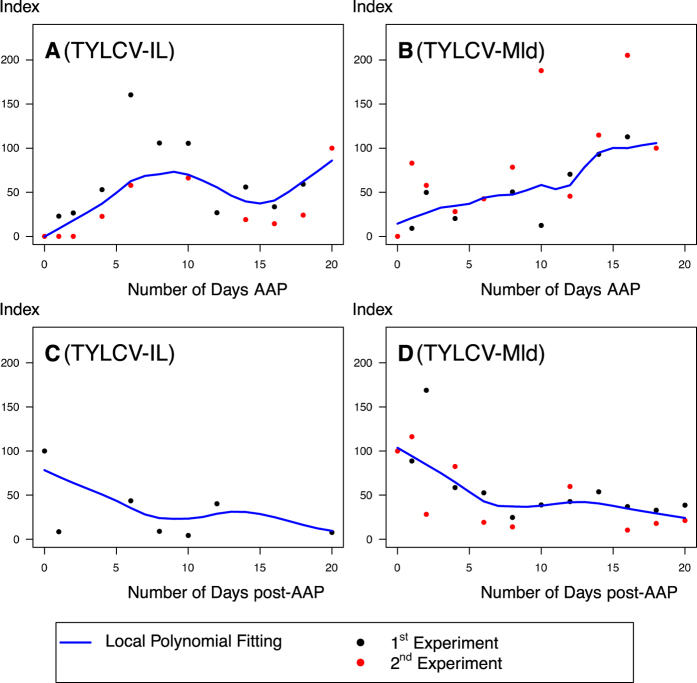
Dynamics of the replicative complementary strand of TYLCV-IL (left panels A, C) and -Mld (right panels B, D) during acquisition access periods (AAP, upper panels A, B) and post-acquisition access periods (post-AAP, lower panels C, D) in MEAM1 *Bemisia tabaci* adults. Groups of 10 pooled whiteflies, collected throughout 20 days AAP or post-AAP (after 3 days AAP) were used in a two-step anchored real-time PCR for the specific quantification of the TYLCV complementary strand. For AAP and post-AAP, arbitrary units (c-strand index) were set at 100% at day 20 and 0, respectively. Each symbol represents the mean value of a duplicate or triplicate. Blue line: local polynomial regression.

Our results show that the complementary DNA strand of TYLCV (for both IL and Mld strains) is present in insects from the first day of AAP, and accumulates progressively over the 20 days of AAP (Fig. 3A,B). The viral complementary strand of TYLCV has recently been shown to accumulate as a component of dsDNA replicative intermediates in plants during the initial steps of infection before reaching a plateau^40^. This complementary strand could allow a minor and transient replication in insects, even though speculative, as suggested in some semi-quantitative studies (transient radionucleotide incorporation until 24 h post-AAP^12^, or two-fold viral load increase until 100 h post-AAP^11^).

On the contrary, the viral complementary strand decreased over the 20 days post-AAP in the insect (Fig. 3C,D). As previously suggested by primer extension followed by Southern blotting, the complementary DNA strand of TYLCV was detected in insects from the first hours following transfer on non-host plant (after 1 h of AAP), until 16 h where it was no more detected^12^. The simplest explanation for the decrease in the quantity of the complementary strands in the insect could be the uptake of TYLCV dsDNA during AAP from the ingested phloem sap, and a progressive decrease post-AAP due to degradation. Supporting this hypothesis, immuno-gold labelling of TYLCV coat protein (CP) in the *B. tabaci* food canal cavity^41^, as well as mutagenesis studies indicating CP-DNA binding and protection^42^, suggest that TYLCV may be ingested from the phloem, not only as a virion (viral strand only), but also as a nucleoprotein including CP and dsDNA. Described for TYLCSV in *N. benthamiana*, dsDNA have been characterised as minichromosomes^43^. Their specific presence in the phloem sap and their possible ingestion by the insect (R.L. Gilbertson, personal communication), as well as their association to proteins such as CP, remain to be demonstrated.

As assessed in *Solanum lycopersicum* at four weeks post-inoculation (as the plants used for the AAP in our study), the TYLCV DNA complementary strand, present within dsDNA molecules at 99%, represents only a minor part (less than 10%) of the total viral strands^40^. Thus the complementary strand (and its subsequent dsDNA form) represents probably just a small proportion of the total viral DNA ingested by *B. tabaci*. Since this complementary strand seems to be progressively degraded after ingestion, without any detectable neo-synthesis (Fig. 3), our results do not argue in favour of substantial TYLCV replication in its insect vector.

### No transovarial transmission of TYLCV DNA and infectivity

One other point that was investigated is transovarial transmission, which can be associated with viral replication in insects^3^, and which has occasionally been reported in MEAM1 *B. tabaci* maintained on tomato plants infected with TYLCV-IL^44^. PCR detection on F1 adults issued from viruliferous females (for TYLCV-IL and TYLCV-Mld) did not produce any evidence of transovarial transmission. Neither TYLCV DNA nor infectivity was associated with the progeny of viruliferous and infectious females of MEAM1 *B. tabaci* (Table S2). Similar results were obtained by Bosco *et al.* (2004) with a Portuguese isolate of TYLCV-IL and the two invasive MEAM1 and MED cryptic species of *B. tabaci*. Moreover, using immunocytochemistry on ovary sections, no specific labelling was observed in eggs of insects fed on plants infected with TYLCSV (a TYLCV-related species)^42^.

## Conclusions

Our results reveal a similar pattern of rapid accumulation and a low degradation rate of both Mld and IL strains of TYLCV in *B. tabaci*. The increase in TYLCV DNA load in its vector during the early days of AAP is due to continuous viral acquisition, and not to viral replication in the insect. Indeed, even if the exponential accumulation of viral DNA and the detection of the viral complementary strand in *B. tabaci* can easily be confused with TYLCV replication in its vector, we did not detect any evidence of replication: neither the viral load nor the replicative forms increased after the end of the viral acquisition access periods, and no vertical transmission was observed. Even if limited replication of TYLCV might take place in its insect vector *B. tabaci*, it is not followed by increasing TYLCV loads post-AAP. Our quantification of the viral loads and of complementary DNA strands of the Mld and IL strains of TYLCV in *B. tabaci* point to an effective accumulation and preservation mechanism, consistent with the transmissible “reservoir” hypothesis of the circulative non-propagative transmission mode. The form in which the virus is stored or transported in the insect vector remains a mystery.

## Methods

### Biological material and experiments

Synchronous MEAM1 *B. tabaci* female adults were given acquisition access periods (AAPs) on tomato plants, cv. Farmer (Known-You Seed), four weeks after their agro-infection with TYLCV-IL or TYLCV-Mld as previously described^22^. The so-called post-acquisition access period (post-AAP) consisted of a transfer of viruliferous insects on cabbage plants (*Brassica oleacera*, cv. Alta, Technisem), which are non-host plants for TYLCV^45^,^46^, in a growth chamber^22^. Thus during post-AAP insects are feeding on a virus non-host plant.

In all the experiments, total DNA was extracted as described elsewhere^47^ and stored at -20 °C until use. Real-time PCR was performed and analysed using the ABI PRISM® 7000 Sequence Detection System (Applied Biosystems®). Triplicate or duplicate values with ΔCt differing by more than 1 unit were discarded, as were PCR experiments with efficiency slopes below -3.59 or above -3.1, corresponding to a PCR efficiency beyond 90% or above 110%.

All the statistical analyses were performed with R, version 2.15.2^48^.

### Within-insect viral quantification: models and estimates of viral accumulation and persistence

#### Twenty days of viral acquisition or post-acquisition access periods

Real-time PCR (performed in duplicate as described previously^49^) was used to quantify TYLCV-Mld (two independent experiments) or TYLCV-IL (one experiment) in groups of 10 pooled insects, which were collected daily and then at two-day intervals during a 20-day AAP, or during a 20-day post-AAP after a 3-day AAP. Additional groups of 10 insects were collected as described above for dissection and further real-time PCR. The head, thorax, and abdomen of the insects were micro-dissected under a binocular microscope with the aid of forceps. To prevent contamination of the hemolymph by the alimentary bolus, the haemolymph was dissolved in a 10 μl drop of sterile physiological saline solution containing 0.2% glucose, brought into contact with a slit made on the ventral side of the abdominal cuticle.

Viral load was estimated by the mean (of the two replicates) of the logarithm of the viral quantity: 
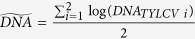
 (where *i* is the replicate index). Then, a linear equation accounting for the strain and experimental effects was used to fit the dynamics of 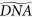, either for acquisition or for persistence. On an arithmetic scale, this equation is equivalent to an exponential model of the DNA load over time.

### Viral acquisition (6 to 48 hours) followed by up to 96 hours of post-acquisition

We developed a duplex real-time PCR procedure to amplify TYLCV simultaneously with the endogenous 18 S gene of *B. tabaci* from individual insects. Primers and probes chosen for *B. tabaci* 18S, as well as the plasmid used to generate the standard curve associated with the 18S gene amplification, are described elsewhere^49^. Primers F1445 (GCCTGAGGAGCAGTGATGAGT) and R1556 (ACCAATAAGGCGTAAGCGTGTAG), with the TaqMan® MGB probe TGTGCGTGAATCCA, designed with PrimerExpress® Software v2.0, were used for quantification of both TYLCV-IL and -Mld. A synthesised oligonucleotide, corresponding to the complementary strand of TYLCV-IL (GenBank: AM409201.1, complementary strand of nucleotides 1556-1445), and 100% identical to the cognate sequence of TYLCV-Mld, was used after PAGE purification to generate the standard curves associated with the amplification of viral targets (GeneCust, Luxembourg).

The duplex real-time PCR was designed to amplify each target (i.e. viral sequence and 18S gene) with similar efficiency whatever the amount of the other target, and used in a final volume of 25 μL: 2 μL of sample extract and 23 μL of mix containing 1X Master Mix (Applied Biosystems®), 267 nM of each 18S primer, 100 nM of Taqman-MGB-VIC 18S probe, 45 nM of F1445, 600 nM of R1556 and 150 nM of Taqman-MGB-FAM TYLCV probe, with 40 cycles of 15 seconds at 95 °C and 45 seconds at 62 °C., following Applied Biosystems guidelines.

The resulting duplex real-time PCR was used to quantify TYLCV DNA in its vector during and after AAP. Two AAP durations were tested, 6 h and 48 h, with both TYLCV-IL and TYLCV-Mld in single infections, resulting in four independent experiments. Each independent experiment was performed twice (in separate cages). Ten individual insects of each strain were collected at 0 h, 6 h for both AAP experiments, and then every 6 to 12 hours for the 48-h AAP experiments. After the insects had been transferred to virus non-host plants, 10 insects were collected every 3 to 12 hours, during post-AAPs of 54 h (for the 6 h AAP experiments) or 96 h (for the 48 h AAP experiments). Each insect was analysed individually by duplex real-time PCR in triplicate.

The simultaneous amplification of the endogenous 18S gene with the viral targets allowed us to calculate a normalised viral load associated with each insect. The normalised viral load was estimated by the mean (of 3 replicates) of the logarithm of the ratio between viral and 18S DNA quantities: 
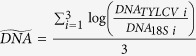
 (where *i* is the replicate index). Next, three models fitting the dynamics of 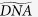
during either the acquisition or the non-acquisition periods were tested by an ANCOVA analysis: 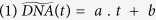; 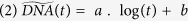; 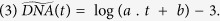


These models correspond respectively to exponential, power, and linear models on an arithmetic scale. The best-fitting models were chosen according to the Akaike information criterion (AIC). These models take into account the effects of the AAP duration, viral strain and insect cage. The term ‘−3’ was added to the linear model to avoid calculating the logarithm of negative values, but did not change the AIC of this model.

### Within-insect specific quantification of the complementary strand

Anchored primers, which classically allow detection of single strands of DNA viruses^50^, were used for TYLCV and TYLCSV *in planta*^40^. We designed a two-step real-time PCR with anchored primers to specifically quantify the TYLCV complementary strand (template of rolling circle replication). The forward TYLCV primer F1445 was anchored at its 5′ end with the unrelated sequence J53, CCCACAAAGAGGCTATGGAA, used in real-time PCR for studies in *Coffea arabica*^51^, and creating no interference with TYLCV amplification. The anchored primer, J53-F1445, was synthesised and purified by PAGE (GenCust, Luxembourg) prior to its use in a first unidirectional PCR, as a way to pre-amplify the complementary strand into products tagged at their 5′ end. This PCR was performed in a final volume of 25 μL for 10 cycles (30 s at 94 °C, 55 °C and 72 °C, following the initial denaturation step at 95 °C), with 1 unit of GoldStar® DNA polymerase (Eurogentech) in 1X associated buffer, 1.5 mM of MgCl_2_, 16 nM of J53-F1445 and 800 nM of dNTPs. A subsequent real-time PCR was performed with 10 μL of the previous amplification, using J53 and R1556 (thus disabling amplification of the viral strand and enabling specific amplification of the complementary strand) in a final volume of 25 μL including 1X Master Mix (Applied Biosystems®), 800 nM of J53, 600 nM of F1445 and 150 nM of Taqman-MGB-FAM TYLCV probe through 40 cycles of 15 seconds at 95 °C and 45 seconds at 62 °C. This anchored-specific real-time PCR was used to quantify the complementary DNA strand of either TYLCV-Mld or TYLCV-IL from the insects collected in the experiment during 20-day AAP or post-AAP, as previously described. The synthesised oligonucleotide used for duplex real-time PCR standards was also used to generate the standard curves associated with the quantification of the TYLCV-IL and -Mld complementary strands, and a synthesised oligonucleotide corresponding to the viral strand was used as negative control (GeneCust, Luxembourg).

### Transovarial transmission assays

A 5-day AAP was performed with MEAM1 *B. tabaci* females on tomato plants agro-inoculated with TYLCV-IL or TYLCV-Mld as previously described^22^. The insects were transferred onto young tomato plantlets for a 1-day inoculation access period (IAP), and then monitored for TYLCV transmission and for the expression of symptoms as described in Perefarres *et al.* (2014)^22^. After transfer onto TYLCV non-host plants (cabbage) for 10 days, the presence of TYLCV DNA was evaluated in the offspring of 12 and 9 viruliferous females (for TYLCV-IL and TYLCV-Mld, respectively) using conventional PCR as described^52^. Finally, the presence of TYLCV DNA in parental females was controlled using conventional PCR^52^ after the 10-day laying period on cabbage.

## Additional Information

**How to cite this article**: Becker, N. *et al.* Rapid accumulation and low degradation: key parameters of *Tomato yellow leaf curl virus* persistence in its insect vector *Bemisia tabaci*. *Sci. Rep.* **5**, 17696; doi: 10.1038/srep17696 (2015).

## Supplementary Material

Supplementary Information

## Figures and Tables

**Table 1:** TYLCV DNA loads during 20 days of acquisition access period (AAP) or post-acquisition access period (post-AAP, following 3 days AAP), in whole insects or dissected insect parts.

Experiment	R^2^	Subdivision	Slope	Log(TYLCV) [CI_95_]
**TYLCV-IL, post-AAP**	0.998	**Whole insects**		**8.32**–**9.05 (a)**
		Abdomen		8.49–9.22 (a)
		Head		7.62–8.37 (b)
		Thorax		7.21–7.94 (bc)
		Haemolymph	>	6.59–7.32 (c)
**TYLCV-Mld, post-AAP,** Exp. 1	0.998	**Whole insects**		**7.84**–**8.40 (a)**
		Abdomen	<	7.34–7.91 (a)
		Thorax		6.73–7.32 (b)
		Haemolymph		5.89–6.46 (c)
		Head		5.15–5.72 (d)
**TYLCV-Mld, post-AAP,** Exp. 2	0.998	**Whole insects**	<	**7.00**–**7.56 (a)**
		Abdomen	<	6.72–7.28 (a)
		Thorax		6.40–6.96 (a)
		Haemolymph		4.66–5.22 (b)
		Head		4.14–4.72 (b)
				
**TYLCV-IL, AAP**	0.999	Abdomen		8.30–8.92 (a)
		Head		6.99–7.61 (b)
		Thorax	>	7.15–7.78 (b)
		Haemolymph	>	5.77–6.42 (c)
**TYLCV-Mld, AAP,** Exp. 1	0.998	Abdomen	>	6.98–7.65 (a)
		Thorax	>	6.98–7.64 (a)
		Haemolymph	>	5.51–6.19 (b)
		Head	>	4.86–5.53 (b)
**TYLCV-Mld, AAP,** Exp. 2	0.999	Abdomen	>	7.01–7.61 (a)
		Thorax	>	6.23–6.83 (b)
		Haemolymph		5.18–5.79 (c)
		Head		4.70–5.32 (d)

Linear regressions of TYLCV loads (from daily duplicate real-time PCRs) were performed during 20 days of AAP or post-AAP. R^2^: adjusted R-squares. Slopes significantly different from 0 (p<0.05) are noted as negative (<) or positive (>). Log(TYLCV): 95% confidence intervals (CI_95_) of virus copy numbers at the intercept. Different lower case letters indicate significant differences (p<0.05).

## References

[b1] Brault V., Uzest M., Monsion B., Jacquot E. & Blanc S. Aphids as transport devices for plant viruses. C. R. Biol. 333, 524–538 (2010). 20541164 10.1016/j.crvi.2010.04.001

[b2] Gray S. M. & Banerjee N. Mechanisms of arthropod transmission of plant and animal viruses Microbiol. Mol. Biol. Rev. 63, 128–148 (1999). 10066833 10.1128/mmbr.63.1.128-148.1999 PMC98959

[b3] Hogenhout S. A., Ammar E.-D., Whitfield A. E. & Redinbaugh M. G. Insect vector interactions with persistently transmitted viruses. Annu. Rev. Phytopathol. 46, 327–359 (2008). 18680428 10.1146/annurev.phyto.022508.092135

[b4] Jeske H. In TT Viruses Vol. 331 Current Topics in Microbiology and Immunology (eds de Villiers Ethel-Michele & Hausen Haraldzur ) Ch. 11, 185–226 (Springer Berlin: Heidelberg, , 2009). 10.1007/978-3-540-70972-5_11 19230564

[b5] De Barro P. J., Liu S. S., Boykin L. M. & Dinsdale A. B. Bemisia tabaci: a statement of species status. Annu. Rev. Entomol. 56, 1–19 (2011). 20690829 10.1146/annurev-ento-112408-085504

[b6] Hanley-Bowdoin L., Bejarano E. R., Robertson D. & Mansoor S. Geminiviruses: masters at redirecting and reprogramming plant processes. Nat. Rev. Microbiol. 11, 777–788 (2013). 24100361 10.1038/nrmicro3117

[b7] Rojas M. R., Hagen C., Lucas W. J. & Gilbertson R. L. Exploiting chinks in the plant’s armor: evolution and emergence of geminiviruses. Annu. Rev. Phytopathol. 43, 361–394 (2005). 16078889 10.1146/annurev.phyto.43.040204.135939

[b8] Diaz-Pendon J. A. *et al.* Tomato yellow leaf curl viruses: menage a trois between the virus complex, the plant and the whitefly vector. Mol. Plant Pathol. 11, 441–450 (2010). 20618703 10.1111/j.1364-3703.2010.00618.x PMC6640490

[b9] Scholthof K. B. *et al.* Top 10 plant viruses in molecular plant pathology. Mol. Plant Pathol. 12, 938–954 (2011). 22017770 10.1111/j.1364-3703.2011.00752.x PMC6640423

[b10] Rubinstein G. & Czosnek H. Long-term association of tomato yellow leaf curl virus with its whitefly vector Bemisia tabaci: effect on the insect transmission capacity, longevity and fecundity. J. Gen. Virol. 78, 2683–2689 (1997). 9349491 10.1099/0022-1317-78-10-2683

[b11] Mehta P., Wyman J. A., Nakhla M. K. & Maxwell D. P. Transmission of tomato yellow leaf curl geminivirus by Bemisia tabaci (Homoptera: Aleyrodidae). J. Econ. Entomol. 87, 1291–1297 (1994). 10.1093/jee/87.5.1285 7962950

[b12] Czosnek H. *et al.* In Adv. Virus Res. 57, 291–322 (Academic Press, 2001). 11680387 10.1016/s0065-3527(01)57006-2

[b13] Sinisterra X. H., McKenzie C. L., Hunter W. B. & Powell C. A. & Shatters, R. G., Jr. Differential transcriptional activity of plant-pathogenic begomoviruses in their whitefly vector (Bemisia tabaci, Gennadius: Hemiptera Aleyrodidae). J. Gen. Virol. 86, 1525–1532 (2005). 15831966 10.1099/vir.0.80665-0

[b14] Ghanim M., Brumin M. & Popovski S. A simple, rapid and inexpensive method for localization of Tomato yellow leaf curl virus and Potato leafroll virus in plant and insect vectors. J. Virol. Methods 159, 311–314 (2009). 19406154 10.1016/j.jviromet.2009.04.017

[b15] Bosco D., Mason G. & Accotto G. P. TYLCSV DNA, but not infectivity, can be transovarially inherited by the progeny of the whitefly vector Bemisia tabaci (Gennadius). Virology 323, 276–283 (2004). 15193923 10.1016/j.virol.2004.03.010

[b16] Wang J. *et al.* Low frequency of horizontal and vertical transmission of two begomoviruses through whiteflies exhibits little relevance to the vector infectivity. Ann. Appl. Biol. 157, 125–133 (2010).

[b17] Pan H. *et al.* Rapid spread of tomato yellow leaf curl virus in China is aided differentially by two invasive whiteflies. PloS one 7(4), e34817, (2012). 22514670 10.1371/journal.pone.0034817 PMC3325912

[b18] Zeidan M. & Czosnek H. Acquisition of tomato yellow leaf curl virus by the whitefly Bemisia tabaci. J. Gen. Virol. 72, 2607–2614 (1991). 1940856 10.1099/0022-1317-72-11-2607

[b19] Ohnishi J., Kitamura T., Terami F. & Honda K.-i. A selective barrier in the midgut epithelial cell membrane of the nonvector whitefly Trialeurodes vaporariorum to Tomato yellow leaf curl virus uptake. J. Gen. Plant Pathol. 75, 131–139 (2009).

[b20] Zang L.-S., Chen W.-Q. & Liu S.-S. Comparison of performance on different host plants between the B biotype and a non-B biotype of Bemisia tabaci from Zhejiang, China. Entomol. Exp. Appl. 121, 221–227 (2006).

[b21] Shi X. *et al.* Bemisia tabaci Q carrying tomato yellow leaf curl virus strongly suppresses host plant defenses. Sci. Rep. 4, 5230 (2014). 24912756 10.1038/srep05230 PMC4050386

[b22] Perefarres F. *et al.* Frequency-dependent assistance as a way out of competitive exclusion between two strains of an emerging virus. Proc. Biol. Sci. 281, (1781) 20133374 (2014). 24598426 10.1098/rspb.2013.3374 PMC3953851

[b23] Akaike H. A new look at the statistical model identification. IEEE Trans. Autom. Control. 19, 716–723 (1974).

[b24] Lett J.-M. *et al.* Spatial and Temporal Distribution of Geminiviruses in Leafhoppers of the Genus Cicadulina Monitored by Conventional and Quantitative Polymerase Chain Reaction. Phytopathology 92, 65–74 (2002). 18944141 10.1094/PHYTO.2002.92.1.65

[b25] Gilbertson R. L. & Lucas W. J. How do viruses traffic on the ‘vascular highway’? Trends Plant Sci. 1, 250–251 (1996).

[b26] Gorovits R., Moshe A., Ghanim M. & Czosnek H. Recruitment of the host plant heat shock protein 70 by Tomato yellow leaf curl virus coat protein is required for virus infection. PLoS One 8 **(7)**, e70280 (2013). 23894631 10.1371/journal.pone.0070280 PMC3720902

[b27] Martinière A. *et al.* A virus responds instantly to the presence of the vector on the host and forms transmission morphs. eLife 2, 00183 (2013). 10.7554/eLife.00183 PMC3552618 23358702

[b28] Mousson L. *et al.* Wolbachia modulates Chikungunya replication in Aedes albopictus. Mol. Ecol. 19, 1953–1964 (2010). 20345686 10.1111/j.1365-294X.2010.04606.x

[b29] Quito-Avila D. F., Lightle D., Lee J. & Martin R. R. Transmission biology of raspberry latent virus, the first aphid-borne reovirus. Phytopathology 102, 547–553 (2012). 22352304 10.1094/PHYTO-12-11-0331

[b30] Cicero J. M. & Brown J. K. Anatomy of Accessory Salivary Glands of the Whitefly Bemisia tabaci (Hemiptera: Aleyrodidae) and Correlations to Begomovirus Transmission. Ann. Entomol. Soc. Am. 104 **(2)**, 280–286 (2011).

[b31] Cicero J. M. & Brown J. K. Ultrastructural Studies of the Salivary Duct System in the Whitefly Vector Bemisia tabaci (Aleyrodidae: Hemiptera). Ann. Entomol. Soc. Am. 105 **(5)**, 701–717 (2012).

[b32] Caciagli P. & Bosco D. Quantitation Over Time of Tomato Yellow Leaf Curl Geminivirus DNA in Its Whitefly Vector. Phytopathology 87, 610–613 (1997). 18945078 10.1094/PHYTO.1997.87.6.610

[b33] Mason G., Caciagli P., Accotto G. P. & Noris E. Real-time PCR for the quantitation of Tomato yellow leaf curl Sardinia virus in tomato plants and in Bemisia tabaci. J. Virol. Methods 147, 282–289 (2008). 17980920 10.1016/j.jviromet.2007.09.015

[b34] Kliot A. & Ghanim M. The role of bacterial chaperones in the circulative transmission of plant viruses by insect vectors. Viruses 5, 1516–1535 (2013). 23783810 10.3390/v5061516 PMC3717719

[b35] Koonin E. V., Mushegian A. R., Ryabov E. V. & Dolja V. V. Diverse groups of plant RNA and DNA viruses share related movement proteins that may possess chaperone-like activity. J. Gen. Virol. 72, 2895–2903 (1991). 1684985 10.1099/0022-1317-72-12-2895

[b36] Gotz M. *et al.* Implication of Bemisia tabaci heat shock protein 70 in Begomovirus-whitefly interactions. J. Virol. 86, 13241–13252 (2012). 23015709 10.1128/JVI.00880-12 PMC3503126

[b37] Uchibori M., Hirata A., Suzuki M. & Ugaki M. Tomato yellow leaf curl virus accumulates in vesicle-like structures in descending and ascending midgut epithelial cells of the vector whitefly, Bemisia tabaci, but not in those of nonvector whitefly Trialeurodes vaporariorum. J. Gen. Plant Pathol. 79, 115–122 (2013).

[b38] Ohnesorge S. & Bejarano E. R. Begomovirus coat protein interacts with a small heat-shock protein of its transmission vector (Bemisia tabaci). Insect Mol. Biol. 18, 693–703 (2009). 19817909 10.1111/j.1365-2583.2009.00906.x

[b39] Gottlieb Y. *et al.* The transmission efficiency of tomato yellow leaf curl virus by the whitefly Bemisia tabaci is correlated with the presence of a specific symbiotic bacterium species. J. Virol. 84, 9310–9317 (2010). 20631135 10.1128/JVI.00423-10 PMC2937599

[b40] Rodriguez-Negrete E. A. *et al.* A sensitive method for the quantification of virion-sense and complementary-sense DNA strands of circular single-stranded DNA viruses. Sci. Rep. 4, 6438 (2014). 25241765 10.1038/srep06438 PMC5377365

[b41] Ghanim M. & Medina V. In Tomato yellow leaf curl virus disease (ed Czosnek Henryk ) Ch. 10, 171–183 (Springer: Netherlands, , 2007).

[b42] Caciagli P. *et al.* Virion stability is important for the circulative transmission of tomato yellow leaf curl sardinia virus by Bemisia tabaci, but virion access to salivary glands does not guarantee transmissibility. J. Virol. 83, 5784–5795 (2009). 19321611 10.1128/JVI.02267-08 PMC2681986

[b43] Paprotka T., Deuschle K., Pilartz M. & Jeske H. Form follows function in geminiviral minichromosome architecture. Virus Res. 196, 44–55 (2015). 25445344 10.1016/j.virusres.2014.11.004

[b44] Ghanim M., Morin S., Zeidan M. & Czosnek H. Evidence for transovarial transmission of tomato yellow leaf curl virus by its vector, the whitefly Bemisia tabaci. Virology 240, 295–303 (1998). 9454703 10.1006/viro.1997.8937

[b45] Stonor J., Hart P., Gunther M., DeBarro P. & Rezaian M. A. Tomato leaf curl geminivirus in Australia: occurrence, detection, sequence diversity and host range. Plant Pathol. 52, 379–388 (2003).

[b46] Mansour A. & Al‐Musa, A. Tomato yellow leaf curl virus: host range and virus‐vector relationships. Plant Pathol. 41, 122–125 (1992).

[b47] Delatte H. *et al.* A new silverleaf-inducing biotype Ms of Bemisia tabaci (Hemiptera : Aleyrodidae) indigenous to the islands of the south-west Indian Ocean. Bull. Entomol. Res. 95 **(01)**, 29–35 (2005). 15705212 10.1079/ber2004337

[b48] Core Team R. (2013). R: A language and environment for statistical computing. R Foundation for Statistical Computing, Vienna, Austria. URL http://www.R-project.org/.

[b49] Perefarres F. *et al.* A novel synthetic quantification standard including virus and internal report targets: application for the detection and quantification of emerging begomoviruses on tomato. Virol. J. 8, 389 (2011). 21819593 10.1186/1743-422X-8-389 PMC3175178

[b50] Jun-Bin S., Zhi C., Wei-Qin N. & Jun F. A quantitative method to detect HBV cccDNA by chimeric primer and real-time polymerase chain reaction. J. Virol. Methods 112, 45–52 (2003). 12951212 10.1016/s0166-0934(03)00190-3

[b51] Joet T., Salmona J., Laffargue A., Descroix F. & Dussert S. Use of the growing environment as a source of variation to identify the quantitative trait transcripts and modules of co-expressed genes that determine chlorogenic acid accumulation. Plant Cell Environ. 33, 1220–1233 (2010). 20199615 10.1111/j.1365-3040.2010.02141.x PMC2904492

[b52] Lefeuvre P., Hoareau M., Delatte H., Reynaud B. & Lett J. M. A multiplex PCR method discriminating between the TYLCV and TYLCV-Mld clades of tomato yellow leaf curl virus. J. Virol. Methods 144, 165–168 (2007). 17485124 10.1016/j.jviromet.2007.03.020

