# Supplementary material for: Rapid accumulation and low degradation: key parameters of Tomato yellow leaf curl virus persistence in its insect vector Bemisia tabaci
Source: Sci Rep. 2015 Dec 2;5:17696. doi: 10.1038/srep17696 (PMC4667217; doi:10.1038/srep17696)
Supplement: Supplementary Information [file srep17696-s1.doc]

**Supplementary information**

**Rapid accumulation and low degradation: key parameters of *Tomato yellow leaf curl virus* persistence in its insect vector *Bemisia tabaci***

Nathalie Becker^1,2 *^*^§^*, Loup Rimbaud^2,3^*^§^*, Frédéric Chiroleu^2^, Bernard Reynaud^2^, Gaël Thébaud^4^, Jean-Michel Lett^2*^

**Figure S1.** **Strain-independent representation of TYLCV DNA load per whitefly *Bemisia tabaci* on an arithmetic scale, during the viral acquisition access period (AAP, in black) and the post-acquisition access period (post-AAP) after 6 h (blue) or 48 h (red) of AAP**. An estimated 196,000 copies were harbored by the insect at the onset of the AAP, and reached mean values of 314,000 after 6 h of AAP and 8.45×10^6^ after 48 h of AAP. After transfer of whiteflies to viral non-host plants (dotted lines), mean values of 56,500 and 5.67×10^6^ viral copies were measured after 6 h and 48 h of AAP, respectively. Note that the apparent decrease in viral load at the onset of post-AAP is not significant (see Table S1 for confidence intervals).


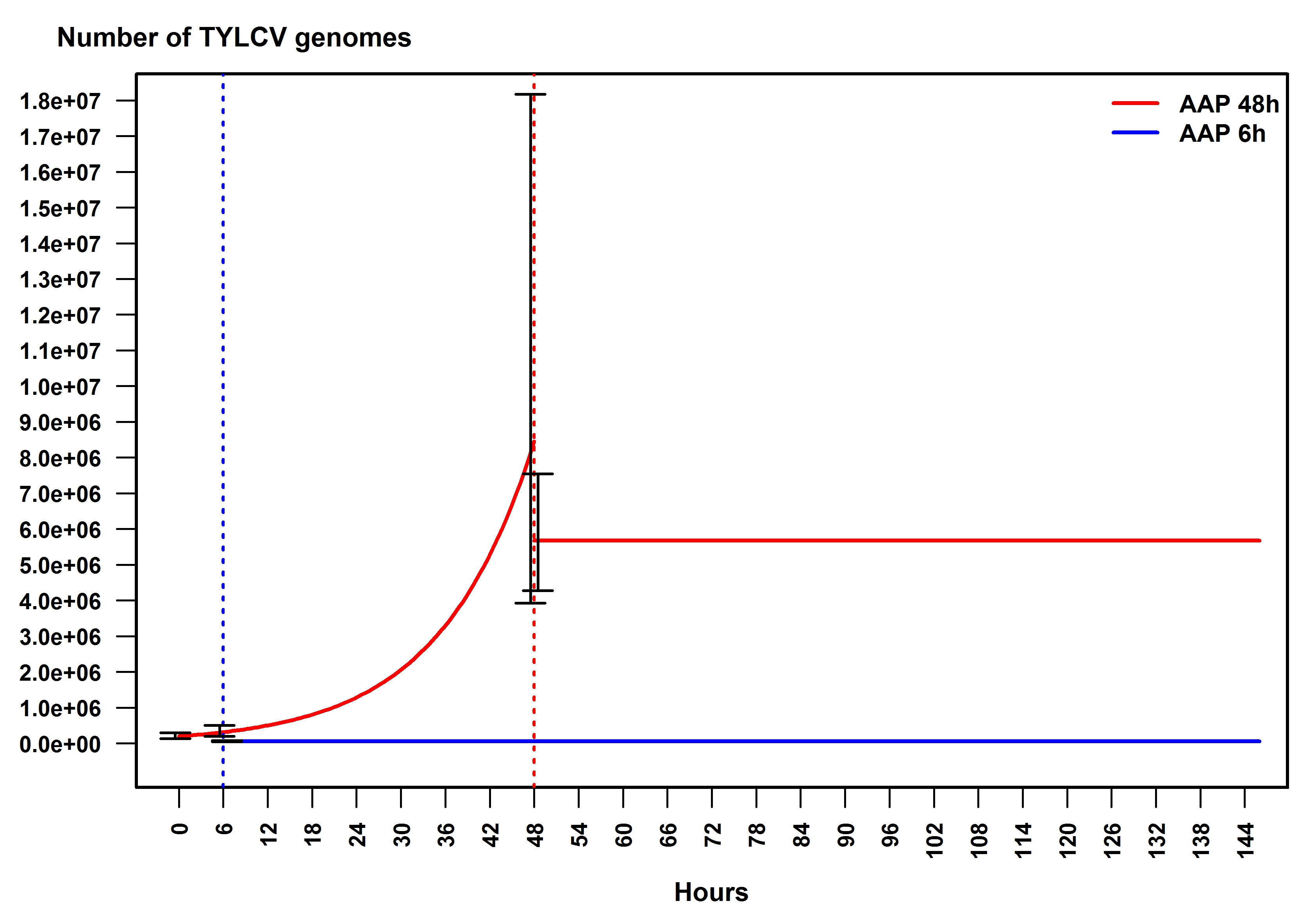


**Table S1**. **Estimation of viral loads in the experiments presented in Fig. 2 and Fig. S1.** Mean value of viral DNA per insect. AAP: acquisition access period. CI: Confidence interval. 0+δt: time just at the beginning of the AAP. Post-AAP: post-acquisition access period.

| **Period** | **AAP duration** | **Time** | **95% CI** | **Mean value** |
| --- | --- | --- | --- | --- |
| **AAP** | 48 h | 0+δt | 0.84×10^5 -^ 4.56×10^5^ | 1.96×10^5^ |
|  |  | 6 h | 1.24×10^5 -^ 7.93×10^5^ | 3.14×10^5^ |
|  |  | 48 h | 1.87×10^6 -^ 38.20×10^6^ | 8.45×10^6^ |
| **Post-AAP** | 48 h | - | 2.83×10^6 -^ 11.37×10^6^ | 5.67×10^6^ |
|  | 6 h | - | 2.75×10^4 -^ 11.62×10^4^ | 5.65×10^4^ |

**Table S2. Results of the transovarial transmission assays in *Bemisia tabaci* with the IL and Mld strains of TYLCV. TYLCV was detected by conventional PCR in offspring of viruliferous and infectious females (12 for TYLCV-IL, 9 for TYLCV-Mld, checked by PCR and transmission tests); TYLCV transmission by the offspring was evaluated based on symptom expression on young tomato plantlets after a 1-day inoculation access period (IAP).**

| TYLCV strain | Viruliferous and  infectious F0 females | TYLCV detection  F1 offspring | TYLCV transmission  F1 offspring |
| --- | --- | --- | --- |
| IL | 12 | 0/67 males 0/63 females | 0/130 |
| Mld | 9 | 0/88 males 0/122 females | 0/210 |
